# Human Monkeypox Outbreak Caused by Novel Virus Belonging to Congo Basin Clade, Sudan, 2005

**DOI:** 10.3201/eid1610.100713

**Published:** 2010-10

**Authors:** Pierre Formenty, Mohammed O. Muntasir, Inger Damon, Vipul Chowdhary, Martin L. Opoka, Charlotte Monimart, Elmangory M. Mutasim, Jean-Claude Manuguerra, Whitni B. Davidson, Kevin L. Karem, Jeanne Cabeza, Sharlenna Wang, Mamunur R. Malik, Thierry Durand, Abdalhalim Khalid, Thomas Rioton, Andrea Kuong-Ruay, Alimagboul A. Babiker, Mubarak E.M. Karsani, Magdi S. Abdalla

**Affiliations:** Author affiliations: World Health Organization Global Alert and Response, Geneva, Switzerland (P. Formenty);; Federal Ministry of Health, Khartoum, Sudan (M.O. Muntasir, A.A. Babiker, M.S. Abdalla);; Centers for Disease Control and Prevention, Atlanta, Georgia, USA (I. Damon, W.B. Davidson, K.L. Karem);; Médecins Sans Frontières France Office in Sudan, Khartoum (V. Chowdhary, C. Monimart, J. Cabeza, T. Durand, A. Khalid, T. Rioton);; World Health Organization Regional Office for the Eastern Mediterranean, Cairo, Egypt (M.L. Opoka);; Minister of Health of Unity State, Bentiu, Sudan (A. Kuong-Ruay);; National Public Health Laboratory, Khartoum (E.M. Mutasim, M.E.M. Karsani);; Institut Pasteur, Paris, France (Jean-Claude Manuguerra);; World Health Organization Country Office, Khartoum (S. Wang, M.R. Malik)

**Keywords:** Monkeypox, orthopoxvirus, epidemiology, outbreak, zoonosis, viruses, human transmission, Sudan, research

## Abstract

To determine the outbreak source of monkeypox virus (MPXV) infections in Unity State, Sudan, in November 2005, we conducted a retrospective investigation. MPXV was identified in a sub-Sahelian savannah environment. Three case notification categories were used: suspected, probable, and confirmed. Molecular, virologic, and serologic assays were used to test blood specimens, vesicular swabs, and crust specimens obtained from symptomatic and recovering persons. Ten laboratory-confirmed cases and 9 probable cases of MPXV were reported during September–December 2005; no deaths occurred. Human-to-human transmission up to 5 generations was described. Our investigation could not fully determine the source of the outbreak. Preliminary data indicate that the MPXV strain isolated during this outbreak was a novel virus belonging to the Congo Basin clade. Our results indicate that MPXV should be considered endemic to the wetland areas of Unity State. This finding will enhance understanding of the ecologic niche for this virus.

## Abstract 2

TOC Summary: This virus should be considered endemic to the wetland areas of Bentiu, Unity State, Sudan.

Monkeypox virus (MPXV) is an orthopoxvirus and the causative agent for human monkeypox, a viral disease with clinical signs in humans similar to those seen in past smallpox patients (*1**,**2*). Human monkeypox is regularly reported in remote villages of central Africa near tropical rainforests where persons may have contact with infected animals (*3**–**7*). In 2003, human monkeypox was identified in the Western Hemisphere (*8*) after importation of rodents from Ghana.

In early November 2005, the medical team at the Médecins Sans Frontières France (MSF-F) hospital in Bentiu, Unity State, Sudan, reported several suspected case-patients with generalized vesicopustular rash that resembled rash caused by MPXV. Biologic samples were collected and sent to the World Health Organization (WHO) Collaborating Center for Smallpox and other Poxvirus Infections, Centers for Disease Control and Prevention (CDC), Atlanta, USA, where MPXV infection was confirmed (*9*). Because human monkeypox had been reported in a dry savannah area in Africa, an investigation team conducted a retrospective analysis of the outbreak. A description of virologic studies indicating that MPXV isolated from the Sudan outbreak is a novel virus in the Congo Basin clade will be detailed in a subsequent report. Here, we report the results of a retrospective investigation that was conducted in January 2006.

## Materials and Methods

Bentiu, capital of Unity State, is situated in southern Sudan ≈750 km southwest of Khartoum, on the southern banks of the Bahr al-Ghazal River (9°14′N, 29°50′E). Unity State is uniformly flat with clay soil and its forests are scattered. During the dry season (November–April) its landscape exhibits characteristics of typical sub-Sahelian savannah; tall grass and inundated swamps predominate during the rainy season of May–October (*10**,**11*).

Unity State was affected greatly during the 21-year civil war in Sudan that largely destroyed the social fabric of its inhabitants (the Nuer), who were seminomadic persons (*10*). Massive displacement of the indigenous population was reported in early 2000; returning refugees and displaced persons from Khartoum, Kenya, and Ethiopia occurred later in 2005. In November 2005, the underlying health infrastructure of Unity State, comprising a network of clinics and healthcare centers and a referral system, was still undergoing construction.

### Initial Report of the Outbreak

On October 27, 2005, the MSF-F hospital team in Bentiu examined an 8-month-old child who had a generalized vesiculopustular rash that was clinically consistent with an orthopoxvirus-associated disease. The child originated from the village of Nuria. He became ill on October 15, 2005, with sudden onset of fever, cough, inflammation of nasal mucous membranes, and enlarged cervical lymph nodes. On October, 16, 2005, a papular rash appeared on the infant’s head and within his mouth. Within 24 hours the rash had spread to cover first his extremities and later his trunk; lesions were also seen on his palms and soles. On October, 29, 2005, a similar disease developed in the child’s mother with abrupt onset of fever and a papular rash, which developed pustular characteristics over the next 5 days. From October 27 through November 18, a preliminary investigation by MSF-F hospital staff identified an additional 18 similar cases in different villages. Crust and blood samples from the mother were sent to CDC in Atlanta, where MPXV infection was confirmed by PCR and virus isolation (*9*). Blood specimens, vesicular swabs, and crust specimens obtained from the 8-month-old child were sent to Institut Pasteur in Paris, where MPXV diagnosis was also confirmed.

### Epidemiologic Surveillance and Investigation of Cases

During January 15, 2006–January 30, 2006, an investigation team, comprising members of the Unity State Ministry of Health (MoH), Sudan Federal MoH, MSF-F, and WHO conducted a retrospective analysis of the MPXV outbreak in Bentiu to identify its source. Three case-notification categories were developed: suspected, probable, and confirmed. A suspected case-patient was defined as any person from the outbreak zone who sought treatment during September 2005–January 2006 for fever (>37.5°C) and vesicular crusty rash. A probable case was defined as any person from the outbreak zone, evaluated by a clinician, who sought treatment during September 2005–January 2006 for fever (>37.5°C), had a vesicular-pustular rash similar to that shown in a WHO reference photograph, and had an epidemiologic link to a confirmed case. If laboratory samples were obtained during or after the illness, then the previous notification categories were reclassified as laboratory-confirmed cases or not a case. Results from a laboratory-confirmed case were considered positive either by ELISA that showed immunoglobulin (Ig) M against orthopoxvirus, PCR amplification of MPXV DNA, or MPXV isolation. Probable cases in which test results were positive by ELISA for IgG and negative for IgM were considered laboratory confirmed if serum samples were collected >56 days after onset of rash and the patient did not have a smallpox vaccination scar or was too young to have been vaccinated against smallpox (*7*). Cases in which test results were negative by PCR and by ELISA for IgG and IgM were classified as not a case.

Suspected case-patients were identified through analysis of medical records from healthcare centers and through passive and active surveillance. Case-patients were passively identified by staff at healthcare and hospital facilities that served as sentinel surveillance sites. Active case finding was organized by the investigation team, who traveled from village to village in areas where suspected cases had been reported. Epidemiologic and clinical information for each case-patient were collected by using data obtained from patient, family, and key informant interviews and from focal group discussions. Data were also obtained by comprehensive review of available patient medical files from the Bentiu MSF-F hospital and other local healthcare centers.

Data for each patient were compiled by using a standardized MPXV investigation form designed to collect information on the identity of the patient, clinical signs, treatment administered during course of disease, potential modes of infection, and laboratory findings. Parameters established by WHO for assessment of smallpox cases (*12*) were used to determine rash severity (i.e., benign, 5–25 lesions; moderate, 26–100 lesions; grave, 101–250 lesions; and extremely grave, >250 lesions). To obtain information on potential modes of infection, investigators sought to determine the range of patient activities during the 2 weeks before illness onset, including visits to healthcare centers or traditional healers, attendance at funeral ceremonies, and any contacts that the patient may have had with other suspected human case-patients or with wild animals.

### Laboratory Analysis and Clinical Specimens

After informed consent was obtained, blood specimens were collected from acute-phase and recovering suspected or probable case-patients; vesicular swabs and crust specimens were collected only from acute-phase case-patients. During October 2005–January 2006, samples (in some cases multiple samples from individual case-patients) were collected from 21 persons: 19 blood samples, 8 vesicular fluid samples, and 7 crust samples. All samples were shipped for analysis to CDC in Atlanta or to Institut Pasteur in Paris.

Molecular, virologic, and serologic assays were used for diagnosis of MPXV. PCR-based molecular assays were performed by using DNA prepared from lesions, swabs, smears, and EDTA–whole blood specimens; in some cases whole blood and dry blood preserved on filter paper were used if no other samples were available. Specimens positive by PCR for MPXV DNA were those that yielded positive results in >2 independent PCR tests (specific for different loci), including 1 that discriminates MPXV-specific DNA signatures from those of other orthopoxviruses. Assays designed to detect generic-level and species-specific DNA signatures and virus culture procedures have been described (*13*). Lesion specimens that did not yield DNA signatures consistent with MPXV were subsequently examined for varicella virus (*14*).

Serologic testing alone was used to define disease status for suspected and probable case-patients who had no active lesions at the time of specimen collection. ELISAs were used for detection of orthopoxvirus-specific IgG or IgM from patient serum samples (*15*). Elevated levels of orthopoxvirus-reactive IgG in serum can indicate either MPXV infection or a previous smallpox vaccination (vaccinia virus) (*7*). Elevated IgM titer (7–56) after onset of rash in persons who had compatible clinical and epidemiologic characteristics was considered confirmation of monkeypox.

## Results

During September 20, 2005–January 31, 2006, a total of 49 cases meeting confirmed, probable, or suspected status definitions were identified in Unity State, Sudan (10 confirmed, 9 probable, and 30 suspected). Among 30 suspected case-patients reported by surveillance, 18 could not be investigated because of logistic issues and 12 were reclassified as non–case-patients after investigation. Among the 12 non–case-patients, 2 who were 50 and 40 years of age were positive for IgG that was attributed to remote vaccination. Other causes of vesicular rash were found among the 12 non–case-patients: cutaneous anthrax (*1*), fungus in HIV patients (*2*), *Staphylococcus* sp (*2*), and chickenpox (*5*).

For epidemiologic analysis, a human monkeypox case was defined as any probable or confirmed case identified during the investigation. All 19 monkeypox case-patients were Nuers, and all recovered from illness (case-fatality rate [CFR] 0%).

Of the 10 laboratory-confirmed case-patients, 3 were PCR positive, 3 were IgM and IgG positive, and 4 were IgG positive and were born after the cessation of the routine smallpox vaccination in 1975 (these 4 case-patients were 5, 11, 13, and 30 years of age, respectively). Tissue from 3 case-patients who were positive for MPXV DNA PCR yielded live virus. The hemagglutinin gene (942 bp) of Sudan viruses was identical to that of the MPXV Congo Basin strain MPXV2003_DRC and MPXV1979_Zaire and had 6 nt changes compared with that of MPXV West Africa strains MPXV2003_US and MPXV_WalterReed267. The full genome sequence comparative analysis is ongoing, but preliminary results show that the MPXV strain isolated during this outbreak has a novel genomic structural variation related to the Congo Basin MPXV clade. All 3 PCR-positive case-patients were from the same village of Nuria; belonged to the same chain of transmission; and had blood specimens, vesicular swabs, and crust specimens obtained 5–13 days after onset of symptoms. Samples were obtained from the 3 confirmed case-patients who were IgM and IgG positive 25–70 days after onset of symptoms. From the 4 confirmed case-patients who were IgG positive, 2 had equivocal tests for IgM and samples were obtained 36 and 39 days after onset of symptoms; 2 were negative for IgM and samples were obtained 50 and 78 days after onset of symptoms.

Of 9 probable case-patients, 2 case-patients agreed to the interview and clinical investigation but declined to provide clinical specimens. The 7 remaining probable case-patients were not available for interviews during the January investigation.

**Figure 1:**
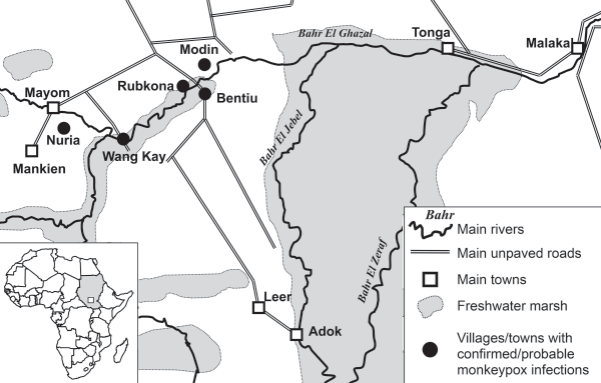
Geographic distribution of cases of human monkeypox virus infection in Unity State, Sudan, 2005. Inset shows location of Sudan (gray shading) and area of consideration within Unity State (white box).

**Figure 2:**
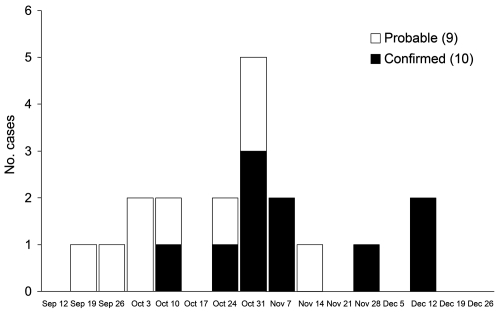
Date of symptom onset for 19 confirmed and probable cases of human monkeypox virus infection in Unity State, Sudan, September 2005–December 2005. Onset date estimated for 5 cases.

The 19 monkeypox case-patients were reported from 5 villages (Figure 1): 2 in Bentiu, 3 in Modin, 5 in Nuria, 5 in Rubkona, and 4 in Wang Kay. All of these villages are located along the herbaceous wetlands along the Bahr-el Ghazal River. The alleged index case-patient, identified retrospectively, became ill on September 20, 2006, in Wang Kay village. The last cases were reported in Rubkona village, with onset of laboratory-confirmed illness occurring on December 15, 2005. The epidemic curve of the outbreak (Figure 2) has a monophasic distribution showing that the peak occurred during October 31, 2005–November 6, 2005. All case-patients were <32 years of age (range 8 months to 32 years), and most (15/19, 79%) were <20 years of age. Ten (52%) of the 19 case-patients were women.

### Description of Different Chains of Transmission

Epidemiologic evidence supported 4 different chains of transmission. Person-to-person transmission was documented in 3 chains. Fourteen case-patients reported contact with a suspected monkeypox case-patient before onset of symptoms; 1 case-patient was probably exposed to infected material during his hospitalization at the MSF-F hospital; and 6 case-patients did not report any known likely mode of infection. Among the 6 case-patients who did not report any known mode of infection, 3 case-patients reported that the rash began appearing around a preexisting wound (1 on the neck, 1 on the foot, and 1 on the arm), which could have been from the bite of an infected animal. None of the monkeypox case-patients reported contact with wild animals.

**Figure 3:**
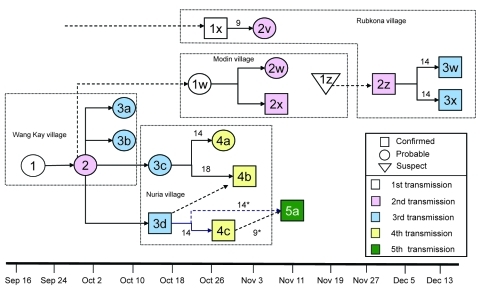
Pattern of virus transmission hypothesized to have occurred during outbreak of human monkeypox in Unity State, Sudan, 2005. Cases are arranged according to date of illness onset in the patient. Solid arrows indicate probable lines of person-to-person transmission; dashed arrows depict undetermined transmission events (e.g., case-patient exposed to persons with monkeypox-like symptoms in same village where no formal link could be established). Numbers near arrows refer to the number of days between case onsets (case intervals); numbers with asterisks (case-patient 5a) refer to interval between possible date of exposure and symptom onset. Dashed boxes enclose case-patients who were living in the same village. Case-patients 1x, 2v, 2w, 2x, 3d, 4c, and 5a were hospitalized in the Médecins Sans Frontières France hospital in Bentiu, Unity State. Three additional cases were not related to these chains of transmission.

Figure 3 shows 3 transmission chains hypothesized to have occurred during the outbreak. The case-patient who we believe may have initiated the transmission chain in Unity State was an 18-year-old man from Wang Kay village. He was unavailable at the time of the investigation for follow-up, and his potential exposure to MPXV was not determined. Local authorities reported that no one was infected before him and that he had had a particularly severe form of the disease; the recovery period lasted several weeks. He may have transmitted the virus to a traditional healer and tooth extractor (case-patient 2) who had treated him and who lived in the same village.

One independent focus of infection without secondary transmission was reported in Bentiu village (1 confirmed case, IgG and IgM positive). This case-patient was a 19-year-old woman who became ill on November 3 and was admitted to the MSF-F hospital on November 10 because of fever and generalized pustular lesions over her entire body. She could not be directly linked to the other chain of transmission, and her mode of infection could not be determined.

### Clinical Characteristics of Case-Patients

**Figure 4:**
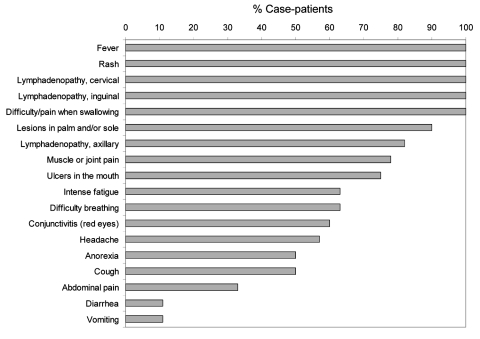
Frequency of individual symptoms reported among human monkeypox virus case-patients in Unity State, Sudan, 2005. Symptoms are arranged from highest to lowest percentage. Note that denominators may vary because confirmed responses were not available from all case-patients.

Of the 19 case-patients, 8 were admitted to the MSF-F Bentiu hospital. The average interval between date of fever onset and date of hospital admission was 8.5 days (median 8.5 days, minimum 4 days, maximum 14 days). The disease progression for 12 patients for whom information was available was similar: a febrile prodrome, cervical lymphadenopathy, throat and joint pain, and later skin eruptions. The median number of days from onset of fever to onset of rash was 4 days (range 1–6 days). Among the 19 case-patients, the most commonly reported signs were fever (16 case-patients), lymphadenopathy (15 case-patients), and maculopapular rash (19 case-patients). Details of the frequency of individual symptoms among the monkeypox case-patients are reported in Figure 4.

Among 11 patients for whom we were able to obtain accurate information on number and location of lesions, 8 had lesions distributed on the entire body and 3 had lesions on the face, arms, and legs. Among the 8 patients who had lesions on the entire body, we obtained information on the number of lesions for 6 of them (3 were moderate, 26–100 lesions; 2 were grave, 101–250 lesions; and 1 extremely grave, >250 lesions). The average and median of case intervals (i.e., the number of days between symptom onsets) was 14 and could be evaluated for 7 couples (case–secondary case).

## Discussion

Smallpox transmission was interrupted in Sudan in 1962, and smallpox did not return until after its reimportation during 1966–1968. The disease spread throughout the country, prompting the establishment of a surveillance and containment program that successfully stopped variola transmission; the last cases were reported in Sudan in December 1972. Mass vaccinations against smallpox continued until 1975, and an international commission certified that Sudan was free of smallpox in November 1978 (*12*). After 33 years of epidemiologic silence, the events reported here from 2005 represent the sole reported outbreak of an orthopoxvirus-associated disease in Sudan in the post-smallpox era.

Despite difficulties during this investigation, we confirmed the presence of human monkeypox disease in Unity State, Sudan. Per our case definition, 10 confirmed and 9 probable cases were reported during September–December 2005. Considering the high number of suspected cases that we were unable to investigate due to logistic constraints and some missing links in several potential transmission chains, we believe the scope of the outbreak is probably underestimated.

Characteristics of the epidemiologic curve are largely compatible with a point source of infection, reinforcing our hypothesis that the traditional healer and dentist (case-patient 2) may have spread the disease to different groups of patients. Young persons were possibly infected while being treated for childhood illnesses, and young adults may have been infected during ritual teeth extraction. No deaths occurred, and evidence of human-to-human transmission for <5 generations was described. We found 4 chains of transmission, 3 of which were associated with the activities of a traditional healer and tooth extractor. Clinical symptoms of case-patients in Sudan was similar to previous descriptions in case-patients from central and West Africa. We found that 6 (75%) of 8 case-patients reported ulcers in the mouth, which is consistent with the frequency of oral lesions reported in unvaccinated patients in the Democratic Republic of the Congo, 1981–1986 (*2*).

In Sudan, as in much of sub-Saharan Africa, extracting incisors (and sometimes canine milk teeth) occurs just after eruption of permanent dentition. This practice is associated with achieving adulthood, beauty, and tribal identity and is necessary for emitting specific linguistic sounds and consuming softer food textures (*16**,**17*). In addition, it is a common belief that canine milk teeth adversely affect the health of infants, causing diarrhea, vomiting, and fever. These teeth are often removed by a traditional healer when symptoms develop in the child. Because these practices are avenues for disease transmission, an educational campaign aimed at reducing the prevalence of this practice should be implemented. Culturally sensitive education could help implement change for safer practices and discourage this harmful ritual practice.

The low CFR observed during this outbreak could not be attributed to treatment, as most case-patients were admitted to the MSF-F hospital very late in the course of disease. Virulence differences between MPXV isolates from West Africa and the Congo Basin have been shown to be caused by genetic differences (*18**,**19*). The low CFR observed in Sudan could be linked to the MPXV circulating in Unity State and its genetic difference with West Africa and Congo Basin isolates. In this regard, our preliminary results showed that the MPXV strain isolated during this outbreak is a novel virus. Alternatively, the low CFR could be related to the transmission of the virus or dose of the virus exposure, and the relatively older ages of affected persons.

This outbreak was reported in an area outside its traditional ecology (tropical rainforests), but rodents that had been incriminated in past human infections in other African countries (e.g., Gambian rat [*Cricetomys gambianus*] or sun squirrels [*Heliosciurius* spp.]) are present in Unity State. This fact suggests that the virus may be endemic to this novel ecologic setting and could have been circulating undetected in nature for a long time. Nonetheless, we did not establish the source of animal-to-human exposure for MPXV.

Regarding the origin of this outbreak, one hypothesis is that the virus may be endemic to the wetland areas of Unity State. This hypothesis is supported by the discovery of a novel MPXV, the low CFR reported in monkeypox case-patients, and the identification of several chains of transmission that had no link between them, implying several introductions into the human population. The suggestion about monkeypox endemicity in Unity State is also supported by several descriptions from villagers; since 2001 similar cases that may be consistent with monkeypox have been recorded in Unity State after severe flooding as was seen during the fall of 2005. Indeed, flooding in Unity Sate during August and September 2005 may have decreased the home range of terrestrial mammals and facilitated the possible contact between potential animal reservoirs and humans. However, several suspected monkeypox cases we investigated were later found to have alternative, laboratory-confirmed diagnoses, thus making anecdotal accounts of possible past human monkeypox outbreaks impossible to confirm as orthopoxvirus infections.

An alternative hypothesis regarding the source of MPXV in Sudan is that the virus was introduced by importation from a neighboring region endemic for MPXV, either by movement of infected animals or through human migration. Still, our investigation did not establish any direct link to either the Democratic Republic of Congo or any of the countries in West Africa where monkeypox is endemic.

Additional investigation, notably ecologic studies, will be needed to demonstrate the endemicity of the virus in Sudan and to discover its natural reservoir host. These studies may have important consequences in the research of the ecologic niche of MPXV (*20*).

After the January 2006 investigation, the MoH in Unity State and MSF-F started a hospital-based surveillance system that enabled detection of 3 case-patients who had suspected pustular rash illnesses. Varicella virus infection was confirmed for all. Unfortunately, surveillance was discontinued in April 2006 because of lack of resources; since then no suspected cases have been reported. To confirm that monkeypox is endemic to Unity State and possibly all of southern Sudan, additional ecologic studies are needed, as well as long-term surveillance for rash and pustular disease, combined with laboratory confirmation of suspected cases.

## References

[1] Ladnyj ID, Ziegler P, Kima E. A human infection caused by monkeypox virus in Basankusu Territory, Democratic Republic of the Congo. Bull World Health Organ. 1972;46:593–7. 4340218 PMC2480792

[2] Jezek Z, Fenner F. Human monkeypox. Monographs in virology. 17th ed. Basel (Switzerland): Karger; 1988.

[3] Heymann DL, Szczeniowski M, Esteves K. Re-emergence of monkeypox in Africa: a review of the past six years. Br Med Bull. 1998;54:693–702. 10326294 10.1093/oxfordjournals.bmb.a011720

[4] Hutin YJ, Williams RJ, Malfait P, Pebody R, Loparev VN, Ropp SL, Outbreak of human monkeypox, Democratic Republic of Congo, 1996 to 1997. Emerg Infect Dis. 2001;7:434–8. 11384521 10.3201/eid0703.010311 PMC2631782

[5] Meyer H, Perrichot M, Stemmler M, Emmerich P, Schmitz H, Varaine F, Outbreaks of disease suspected of being due to human monkeypox virus infection in the Democratic Republic of Congo in 2001. J Clin Microbiol. 2002;40:2919–21. 10.1128/JCM.40.8.2919-2921.2002 12149352 PMC120683

[6] Rimoin AW, Kisalu N, Kebela-Ilunga B, Mukaba T, Wright L, Formenty P, Endemic human monkeypox, Democratic Republic of Congo, 2001–2004. Emerg Infect Dis. 2007;13:934–7. 17553242 10.3201/eid1306.061540 PMC2792850

[7] Learned LA, Reynolds MG, Wassa DW, Li Y, Olson VA, Karem K, Extended interhuman transmission of monkeypox in a hospital community in the Republic of the Congo, 2003. Am J Trop Med Hyg. 2005;73:428–34. 16103616

[8] Reed KD, Melski JW, Graham MB, Regnery RL, Sotir MJ, Wegner MV, The detection of monkeypox in humans in the Western Hemisphere. N Engl J Med. 2004;350:342–50. 10.1056/NEJMoa032299 14736926

[9] Damon IK, Roth CE, Chowdhary V. Discovery of monkeypox in Sudan. N Engl J Med. 2006;355:962–3. 10.1056/NEJMc060792 16943415

[10] Evans-Pritchard EE. The Nuer: a description of the modes of livelihood and political institutions of a Nilotic people. New York: Oxford University Press; 1969.

[11] Hutchinson SE. Nuer dilemmas: coping with money, war, and the state. Berkeley (CA): University of California Press; 1996.

[12] Fenner F, Henderson DA, Arita I, Jezek Z, Ladnyi ID. Smallpox and its eradication. Geneva (Switzerland): World Health Organization; 1988.

[13] Li Y, Olson VA, Laue T, Laker MT, Damon IK. Detection of monkeypox virus with real-time PCR assays. J Clin Virol. 2006;36:194–203. Epub 2006 May 30. 10.1016/j.jcv.2006.03.012 16731033 PMC9628957

[14] Loparev VN, McCaustland K, Holloway BP, Krause PR, Takayama M, Schmid DS. Rapid genotyping of varicella-zoster virus vaccine and wild-type strains with fluorophore-labeled hybridization probes. J Clin Microbiol. 2000;38:4315–9. 11101557 10.1128/jcm.38.12.4315-4319.2000 PMC87598

[15] Karem KL, Reynolds M, Braden Z, Lou G, Bernard N, Patton J, Characterization of acute-phase humoral immunity to monkeypox: use of immunoglobulin M enzyme-linked immunosorbent assay for detection of monkeypox infection during the 2003 North American outbreak. Clin Diagn Lab Immunol. 2005;12:867–72. 16002637 10.1128/CDLI.12.7.867-872.2005 PMC1182207

[16] Willis MS, Schacht RN, Toothaker R. Anterior dental extractions among Dinka and Nuer refugees in the United States: a case series. Spec Care Dentist. 2005;25:193–8. 10.1111/j.1754-4505.2005.tb01649.x 16295224

[17] Wahab AMM. Traditional practice as a cause of infant morbidity and mortality in Juba area (Sudan). Ann Trop Paediatr. 1987;7:18–21. 2438998 10.1080/02724936.1987.11748467

[18] Chen N, Li G, Liszewski MK, Atkinson JP, Jahrling PB, Feng Z, Virulence differences between monkeypox virus isolates from West Africa and the Congo Basin. Virology. 2005;340:46–63. 10.1016/j.virol.2005.05.030 16023693 PMC9534023

[19] Huhn GD, Bauer AM, Yorita K, Graham MB, Sejvar J, Likos A, Clinical characteristics of human monkeypox, and risk factors for severe disease. Clin Infect Dis. 2005;41:1742–51. Epub 2005 Nov 11. 10.1086/498115 16288398

[20] Levine RS, Peterson AT, Yorita KL, Carroll D, Damon IK, Reynolds MG. Ecological niche and geographic distribution of human monkeypox in Africa. PLoS ONE. 2007;2:e176. 10.1371/journal.pone.0000176 17268575 PMC1769466

